# Uncovering the mechanisms of MuRF1-induced ubiquitylation and revealing similarities with MuRF2 and MuRF3

**DOI:** 10.1016/j.bbrep.2023.101636

**Published:** 2024-01-06

**Authors:** Samuel O. Lord, Peter W.J. Dawson, Jitpisute Chunthorng-Orn, Jimi Ng, Leslie M. Baehr, David C. Hughes, Pooja Sridhar, Timothy Knowles, Sue C. Bodine, Yu-Chiang Lai

**Affiliations:** a School of Sport, Exercise and Rehabilitation Sciences, University of Birmingham, Birmingham, UK; b MRC Versus Arthritis Centre for Musculoskeletal Ageing Research, University of Birmingham, Birmingham, UK; c Division of Endocrinology and Metabolism, Department of Internal Medicine, Carver College of Medicine, University of Iowa, Iowa City, IA, USA; d School of Biosciences, University of Birmingham, Birmingham, UK

**Keywords:** RING E3 Ligase, Ubiquitin Conjugating Enzyme (UBE2), Ubiquitin, Autoubiquitylation, In vitro, Muscle Atrophy

## Abstract

MuRF1 (Muscle-specific RING finger protein 1; gene name TRIM63) is a ubiquitin E3 ligase, associated with the progression of muscle atrophy. As a RING (Really Interesting New Gene) type E3 ligase, its unique activity of ubiquitylation is driven by a specific interaction with a UBE2 (ubiquitin conjugating enzyme). Our understanding of MuRF1 function remains unclear as candidate UBE2s have not been fully elucidated. In the present study, we screened human ubiquitin dependent UBE2s in vitro and found that MuRF1 engages in ubiquitylation with UBE2D, UBE2E, UBE2N/V families and UBE2W. MuRF1 can cause mono-ubiquitylation, K48- and K63-linked polyubiquitin chains in a UBE2 dependent manner. Moreover, we identified a two-step UBE2 dependent mechanism whereby MuRF1 is monoubiquitylated by UBE2W which acts as an anchor for UBE2N/V to generate polyubiquitin chains. With the in vitro ubiquitylation assay, we also found that MuRF2 and MuRF3 not only share the same UBE2 partners as MuRF1 but can also directly ubiquitylate the same substrates: Titin (A168-A170), Desmin, and MYLPF (Myosin Light Chain, Phosphorylatable, Fast Skeletal Muscle; also called Myosin Light Regulatory Chain 2). In summary, our work presents new insights into the mechanisms that underpin MuRF1 activity and reveals overlap in MuRF-induced ubiquitylation which could explain their partial redundancy in vivo.

## Highlights

• MuRF1 engages in ubiquitylation with four UBE2 families.
• UBE2W and UBE2N/V function cooperatively during MuRF1 and substrate ubiquitylation.
• All MuRF E3 ligases function with the same UBE2 enzymes in vitro.
• All MuRF E3 ligases can ubiquitylate Titin, MYLPF and Desmin in vitro.

## 1 Introduction

MuRF1 (Muscle-specific RING finger protein 1) plays a critical role in skeletal muscle atrophy. Many studies in humans and rodents, demonstrate that MuRF1 gene (TRIM63) and protein expression increase following numerous atrophy models including disuse, denervation, cancer, renal failure, heart failure, burn injury, fasting, diabetes, corticosteroid treatment and cytokine exposure [[1], [2], [3], [4], [5], [6], [7]] Importantly, suppression of MuRF1 expression perturbs atrophy induced by denervation, glucocorticoid treatment, limb unloading, and lung injury [2,[8], [9], [10]]. These earlier studies have established MuRF1 as a key regulator of skeletal muscle mass. Nonetheless, insight into the molecular mechanisms of MuRF1-induced muscle atrophy is unclear, slowing the development of therapeutics that target MuRF1-induced muscle atrophy.

MuRF1 is an E3 ligase, which functions to target proteins for ubiquitylation. The process of protein ubiquitylation is a coordinated sequence of three enzymatic actions: by an E1 ubiquitin-activating enzyme (UBE1), an E2 ubiquitin-conjugating enzyme (UBE2), and finally an E3 ubiquitin-ligase. The UBE1 enzyme hydrolyses ATP to adenylate ubiquitin, which is transferred to an UBE2 active site. As MuRF1 is a RING-type (Really Interesting New Gene) E3 ligase, with no catalytic activity, it requires the interaction of a UBE2 to directly transfer ubiquitin to the substrate(s) [11]. UBE2s are responsible for the type of ubiquitylation (mono-, multi-mono-, or poly-ubiquitylation) and the structure of ubiquitin chains based on ubiquitin-ubiquitin attachment residue [12,13]. There are eight different polyubiquitin chain types (M1, K6, K11, K27, K29, K33, K48 and K63) and the topology of ubiquitin chain types ultimately determine the fate of the target protein, such as degradation, localisation, or other signalling events [14]. Therefore, to understand the functional role of MuRF1 ubiquitylation onto its substrate, one must identify the UBE2 that partners with MuRF1. Identifying MuRF1 partnering UBE2s would also provide a tool to directly explore substrates of MuRF1 in vitro and characterise their specific form of ubiquitylation.

Previous studies have attempted to identify UBE2s that interact with MuRF1. Polge et al. [15] applied yeast two-hybrid screen and SPR (surface plasmon resonance) technologies and identified several UBE2s, including UBE2E1, UBE2G1, UBE2J1, UBE2J2, and UBE2L3, as interacting with MuRF1. However, these two methods only measure protein-protein interaction without detecting ubiquitin E3 ligase activity of MuRF1. When MuRF1-UBE2 ubiquitylation activity has been studied using ELISA based methods, only 11 UBE2s have been explored [16]. Due to limited characterisation of MuRF1-UBE2 partners, studies have often used UBE2D (UBCH5) family when investigating MuRF1 substrates [[17], [18], [19], [20]]. While this offers some insights into MuRF1-UBE2D ubiquitylation activity, it is worth noting that the UBE2D family may be promiscuous and can interact and produce ubiquitylation activity with most RING type E3 ligases [13]. Therefore, the current literature offers limited understanding of MuRF1-UBE2 partners and how they relate to MuRF1 ubiquitylation function. A study of all human UBE2s with MuRF1 is necessary to further understand the mechanism of MuRF1-mediated ubiquitylation.

MuRF1 shares high homology with two other TRIM family members, MuRF2 (TRIM55) and MuRF3 (TRIM54). Sequence alignment shows MuRF1 displays 62 % and 77 % overlap with MuRF2 and MuRF3 respectively [21]. Given their similar sequence it is possible that they share overlapping roles, a concept supported by genetic mouse models which highlight the redundancy of MuRF E3 ligases. For example, removal of MuRF1 has no detrimental effect on mice phenotype [2,20,22]. However, when MuRF2 or MuRF3 are also removed this causes severe detrimental effects on skeletal and cardiac muscle size and function. Double knockout (dKO) of MuRF1 and MuRF2 causes hypertrophic cardiomyopathy that resulted in the death of ∼75 % of mice in their first few weeks [23,24]. MuRF1 and MuRF3 dKO mice experience skeletal muscle myopathy and hypertrophic cardiomyopathy [20]. Similar features were observed in human patients with mutations in MuRF1 (homozygous) and MuRF3 (heterozygous) [25]. These findings suggest that the loss of MuRF1 can be somewhat compensated by the presence of MuRF2 or MuRF3. Given the lack of molecular understanding surrounding MuRF-induced ubiquitylation, there is no comprehensive explanation for this response. Therefore, a study directly comparing MuRF1, MuRF2 and MuRF3 ubiquitylation is needed.

In the present study a full human UBE2 library (excluding ubiquitin-like UBE2s; see Table S1) were screened using a standard in vitro ubiquitylation assay. This revealed that UBE2D, UBE2E, UBE2N/V families and UBE2W partner with MuRF1 during ubiquitylation. We found that MuRF1 partners with UBE2W and UBE2N/V in a sequential two-step fashion, forming K63-linked ubiquitin chains. Moreover, we showed that MuRF2 and MuRF3 also function with these UBE2s during ubiquitylation and can target the same set of substrates as MuRF1 (Titin, Desmin and MYLPF), providing a molecular explanation for their functional redundancy in vivo.

## 2 Materials and methods

### 2.1 Constructs

MBP-MuRF1, MBP-MuRF2, MBP-MuRF3, Ubiquitin, HIS-UBE1 and the full library of human E2s were sourced from the Medical Research Council - Protein, Phosphorylation and Ubiquitylation Unit (MRC PPU) Reagents and Services (https://mrcppureagents.dundee.ac.uk/) (Table S3). Plasmids for HIS-Titin were provided by Prof. Olga Mayans (University of Konstanz). Titin was cloned into a pMEX3Cb vector to express His-Titin. Recombinant Desmin (A60041) and His-SUMO-MYLPF (A225264) were bought from antibodies.com.

### 2.2 Expression and purification of proteins

Plasmids were transformed into BL21 competent Escherichia Coli (E. Coli) cells. A single colony was selected and inoculated in Ampicillin-treated LB media expanding to 1 L (For MBP-MuRF1, MBP-MuRF2 and MBP-MuRF3 expression 200 μM ZnSO4 was also added prior to Isopropyl β-d-1-thiogalactopyranoside (IPTG) induction) at 37 °C 180 rpm. Bacteria were grown to OD 600 of 0.6 before the addition of 250 μM IPTG to induce protein expression. Growth was inhibited by reducing the temperature down to 18 °C and left overnight to continue protein expression. The cells were pelleted by centrifugation at 5000×*g* at 4 °C for 15 min. Pellets were resuspended in lysis buffer (HIS tag: 50 mM Tris-HCl pH 8.0, 150 mM NaCl, 50 mM Imidazole, 0.5 mM TCEP, 1 mM PMSF. MBP tag: 50 mM Tris-HCl pH 7.5, 150 mM NaCl, 5 % Glycerol, 1 mM TCEP, 1 mM PMSF) and lysed using an Emulsiflex C3 Cell Disruptor (Avestin Europe, Mannheim, Germany). Lysate was cleared at 5000×*g* 4 °C for 2 h and filtered using a 0.45 μm filter to remove any remaining cell debris. Recombinant proteins were purified using His-Trap (GE Healthcare) or Amylose resin (New England Biosciences) as per manufacturer's protocol. Protein purity was confirmed using a Coomassie blue stain (Thermo Fisher Scientific) and protein concentration was determined using a nanodrop. Protein samples were concentrated using 50 kDa centrifugal filters (Amicon, Merck) and stored at −80 °C.

### 2.3 In vitro ubiquitylation assay

In vitro reaction (50 μl) contained 50 mM HEPES pH 7.5, 1 mM DTT, 10 mM MgCl2, 1 mM ATP, 50 μg ubiquitin, 0.2 μg HIS-UBE1, 0.6 μg UBE2 and 2.5 μg MBP-MuRF1, MBP-MuRF2 or MBP-MuRF3. For experiments involving substrates (His-Titin fragment (A168-A170), Desmin or His-SUMO-MYLPF), 0.5 μg of substrate was included alongside 0.5 μg of MBP-MuRF1, MBP-MuRF2 or MBP-MuRF3. Reactions were performed at 37 °C for 1 h at 1000 rpm on the Thermoshaker (Eppendorf) and terminated with the addition of 4x LDS sample buffer (Thermo Fisher Scientific) containing 5 % β-mercaptoethanol to final concentration 1x and 1.25 % respectively. Samples were left overnight at room temperature to denature.

### 2.4 Western blotting

Samples were loaded on 8 % acrylamide Bis-Tris gels and separated using SDS-PAGE gel electrophoresis. Gels were run in 1x MOPS buffer for approximately 90 min at 140 V. Proteins were transferred onto PVDF membranes (Millipore, Hertfordshire, UK) for 2.5 h at 30 V based on an optimised protocol for detecting polyubiquitin chains [26]. Membranes were blocked in 5 % BSA diluted in Tris-buffered saline Tween-20 (TBS-T) for 1 h and incubated overnight at 4 °C with the appropriate primary antibody: Anti-MBP (E8038S, 1:60,000) from New England Biolabs, 6xHis (631212, 1:10,000) from Clontech, Anti-MYLPF (MF-5, 1:1000) from Developmental Studies Hybridoma Bank, Desmin (5332, 1:1000) from Cell Signalling Technology, Anti-ubiquitin P4D1 (646302, 1:1000) from BioLegend, Anti-ubiquitylated proteins FK2 (04–263, 1:1000), Anti-Ubiquitin K48-Specific (05–1307, 1:1000) and Anti-Ubiquitin K63-Specific (05–1313, 1:1000) from Merck-Millipore. Membranes were washed in TBS-T three times prior to 1 h incubation at room temperature in horseradish peroxidase-conjugated secondary antibodies from Cell Signalling Technology (1:10,000). Membranes were washed a further three times in TBS-T prior to antibody detection using enhanced chemiluminescence horseradish peroxidase substrate detection kit (Millipore, Hertfordshire, UK). Imaging was undertaken using a G:BOX Chemi-XR5 (Syngene, Cambridgeshire, UK).

### 2.5 Study approval for animal experiments

The mice used in these studies were males from the C57BL/6 strain obtained from Charles River Laboratories at ages 3–4 month and used for experiments within 2 weeks of their arrival. Animals were housed in ventilated cages maintained in a room at 21 °C with 12-h light/dark cycles and had ad libitum access to standard chow (Harlan-Teklad formula 7913) and water throughout the study. Using the same samples obtained from previous published experiments [27], we undertook new analysis of UBE2 gene expression. Briefly, denervation-induced skeletal muscle atrophy was performed as previously described [27,28], through targeted denervation of the lower limb muscles of the right leg was accomplished via transection of the sciatic nerve in the midthigh region of mice using forceps. The procedure was completed under isoflurane anaesthesia (3 % inhalation) with the use of aseptic surgical techniques. Mice were given an analgesic (buprenorphine, 0.1 mg kg^−1^) immediately, as well as for 48 h following surgery, and returned to their cage following recovery. Following completion of the appropriate time period (3, 7, 14, and 21 days; *n* = 6 per group), mice were anaesthetized with 3 % isoflurane, and the gastrocnemius complex (GSTC) muscles were excised, weighed, frozen in liquid nitrogen and stored at −80 °C for later analysis. On completion of tissue removal, mice were killed by exsanguination. A separate untreated cohort of animals (*n* = 6) was used as the relevant control. All animal procedures were approved by the Institutional Animal Care and Use Committee at the University of Iowa.

### 2.6 RNA isolation and qPCR

RNA isolation and qPCR were performed as previously described [29]. RNA was isolated from frozen gastrocnemius muscle powder using RNAzol RT reagent (Sigma‐Aldrich, St Louis, MO). Complementary DNA (cDNA) was generated from 1 μg of RNA using the iScript Reverse Transcription Supermix kit (BioRad, Hercules, CA). qPCR experiments were performed using the Power SYBR Green master mix (Thermo Fisher Scientific) on a Quantstudio 6 Flex Real‐time PCR System (Applied Biosystems, Foster City, CA). Gene expression values were normalised to muscle mass, as previously performed [[29], [30], [31]]. The mouse primers used in this study are shown in Table S2.

### 2.7 Statistical analysis

Data presented as ± SEM. The statistical analyses were performed using Prism (GraphPad Software). One-way ANOVA was performed with Tukey's post hoc test and p values < .05 were considered statistically significant.

## 3 Results

### 3.1 MuRF1 interacts with UBE2D, E, N/V families, and UBE2W to induce ubiquitylation

**Fig. 1:**
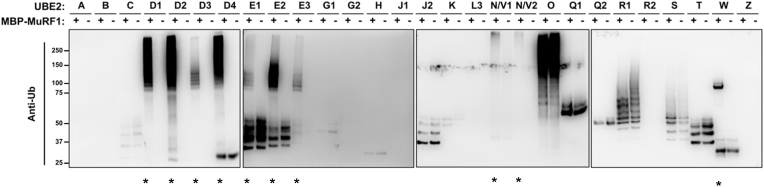
**In vitro ubiquitylation assay shows UBE2D, UBE2E, UBE2N/V, and UBE2W family members partnering with MuRF1 to form conjugated ubiquitin (mono- or poly-ubiquitin).** A library of 28 human ubiquitin E2s (with exception to non-classical Ubiquitin E2s - listed in Table S1) were incubated with or without MBP-MuRF1 for 1 h during an in vitro ubiquitylation assay. Samples were subject to SDS-PAGE gel electrophoresis before *w*estern blot imaging to detect MuRF1-dependent ubiquitylation using anti-ubiquitylated proteins antibody. UBE2s that form MuRF1-dependent ubiquitylation are highlighted with an asterisk (*).

To investigate which UBE2s function with MuRF1, a full screen of 28 recombinant human UBE2s was undertaken to determine which catalyse MuRF1-dependent ubiquitylation in vitro. It is known that some UBE2s can build ubiquitin chains without the need of an E3 ligase, therefore we screened UBE2s with and without MuRF1 to detect MuRF1-dependent ubiquitylation. The results showed that MBP-MuRF1 interacts with UBE2D family (D1, D2, D3 and D4), UBE2E family (E1, E2, and E3), UBE2N/V1 and UBE2N/V2 by forming polyubiquitin chains (Fig. 1). We found that UBE2W monoubiquitylates MuRF1, illustrated by the single band ∼90 kDa (Fig. 1). To ensure that ubiquitylation occurs on MuRF1 and not the MBP tag, we performed an in vitro ubiquitylation assay with either MBP-MuRF1 or MBP-alone which showed that ubiquitylation did not occur on MBP tag (Fig. S1). Some UBE2s can form unanchored ubiquitin chains [32], depicted by ubiquitin bands below the molecular weight of MBP-MuRF1 (∼85 kDa). Overall, this data demonstrates MuRF1 produces distinct ubiquitylation in a UBE2 dependent manner.

### 3.2 MuRF1 autoubiquitylates by a sequential interaction with UBE2W then UBE2N/V family to form K63-linked polyubiquitin chains

**Fig. 2:**
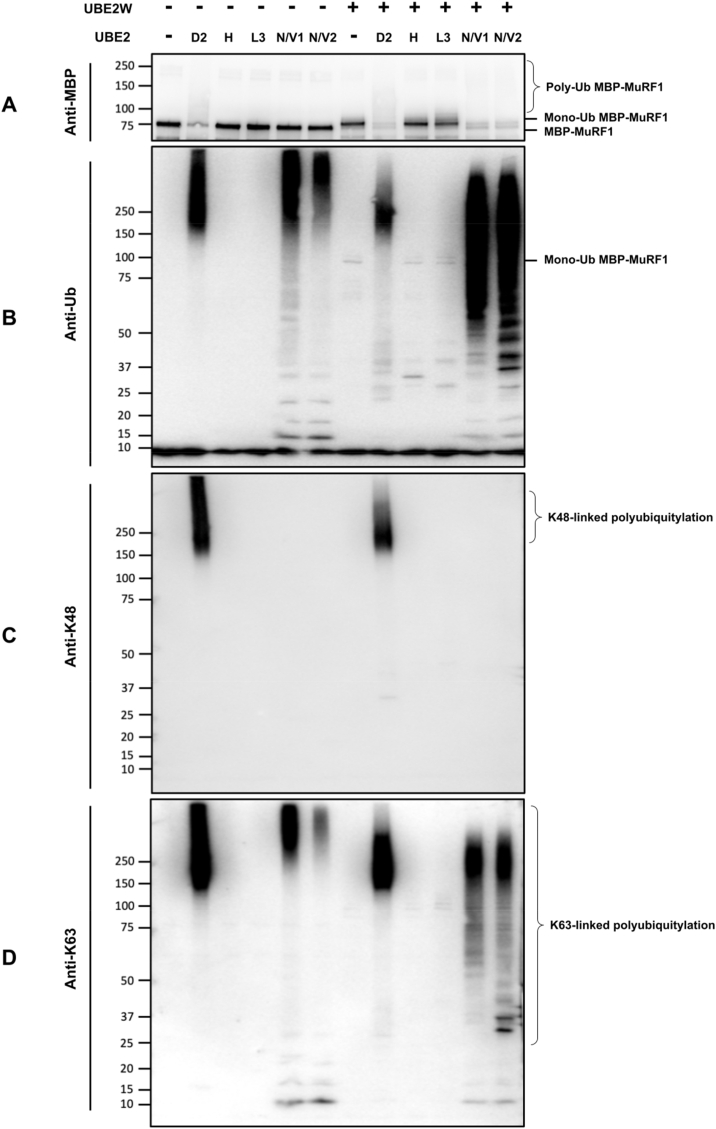
**Combination of UBE2W with other MuRF1-interacting UBE2s shows that UBE2W and UBE2N/V cooperate to generate MuRF1-anchored K63 polyubiquitin chains.** In vitro ubiquitylation assay of MuRF1-interacting E2s: UBE2D2, N/V1 and N/V2, were incubated in the presence or absence of UBE2W. UBE2H and UBE2L3 were used as negative controls. The reaction mixtures were separated by SDS-PAGE and proteins detected by anti-MBP (A), anti-ubiquitin (B), anti-ubiquitin K48-Specific (C) and anti-ubiquitin K63-Specific (D).

Previous research has shown other TRIM E3 ligases, TRIM5ɑ and TRIM21, partner with UBE2W to form monoubiquitin as an anchor to attach additional polyubiquitin chains [32,33]. As such, we proceeded to explore whether this mechanism also occurs with MuRF1. First, we determined which UBE2s were capable of attaching ubiquitin onto MuRF1 itself (autoubiquitylation). We found that unlike the other MuRF1 partnering UBE2s, UBE2N/V1 and UBE2N/V2 generated polyubiquitin chains unbound to MBP-MuRF1 (Fig. S2). This is evident from the level of unmodified MBP-MuRF1, which is dramatically reduced in the presence of UBE2D, E and W, but unchanged by UBE2N/V. Therefore, we hypothesised that the UBE2N/V family requires UBE2W to form anchored polyubiquitin chains. To test this hypothesis, we examined the ubiquitin chains formed by each MuRF1-interacting UBE2 in the presence or absence of UBE2W. We found that the combination of UBE2N/V1 or N/V2 with UBE2W causes polyubiquitylation of MBP-MuRF1 (Fig. 2A). This indicates that monoubiquitin is an anchor on MuRF1 to allow further polyubiquitin chains, generated by UBE2N/V1 or N/V2, to attach. The polyubiquitin chains formed by UBE2D2 shifted to a lower molecular weight in the presence of UBE2W, suggesting the ubiquitin chain lengths were shortened (Fig. 2B). Therefore, whilst we can confirm that MuRF1 autoubiquitylation by UBE2D family is not UBE2W dependent, we cannot rule that UBE2W alters the topology of ubiquitin chains. Furthermore, probing for specific polyubiquitin chain types demonstrated that UBE2N/V1 and N/V2 can generate K63-linked, but not K48-linked, polyubiquitin chains (Fig. 2C and D). Consistent with previous experiments, UBE2D2 was able to form polyubiquitin chains on MBP-MuRF1 (Fig. 2A and B). UBE2D families are able to generate all eight different linkage types of polyubiquitin chains [34]. In similar fashion UBE2D2 was able to generate K48- and K63-linked polyubiquitin chains (Fig. 2C and D).

### 3.3 MuRF2 and MuRF3 partner with the same UBE2s as MuRF1 to execute protein ubiquitylation

**Fig. 3:**
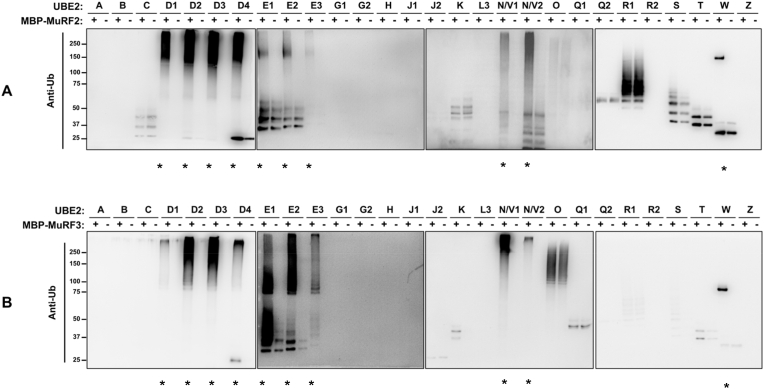
**In vitro ubiquitylation assay shows UBE2D, UBE2E, UBE2N/V, and UBE2W family members partnering with MuRF2 and MuRF3 to form conjugated ubiquitin (mono- or polyubiquitin).** 28 different UBE2s were incubated with or without a) MBP-MuRF2 or b) MBP-MuRF3 for 1 h during an in vitro ubiquitylation assay. Samples were subject to SDS-PAGE gel electrophoresis before western blot imaging to detect MuRF2- and MuRF3-dependent ubiquitylation using anti-ubiquitylated proteins antibody. UBE2s that form MuRF2- and MuRF3-dependent ubiquitylation are highlighted with an asterisk (*).

Sequencing of each MuRF E3 ligase shows over 80 % alignment across their N-terminal residues, including the UBE2-binding RING finger domain [21]. Therefore, we hypothesised that MuRF1, MuRF2 and MuRF3 would share UBE2 partners. To investigate this, we screened MuRF2 and MuRF3 with 28 UBE2s to determine whether they partner with the same UBE2s as MuRF1. We found that as with MuRF1, MuRF2 and MuRF3 also function with the UBE2D, UBE2E, UBE2N/V and UBE2W family (Fig. 3). As expected, UBE2W attached a single ubiquitin molecule to MBP-MuRF2 and MBP-MuRF3, whereas UBE2D, UBE2E, and UBE2N/V produced polyubiquitin chains.

### 3.4 MuRF E3 ligases directly ubiquitylates Titin, MYLPF and Desmin in vitro

**Fig. 4:**
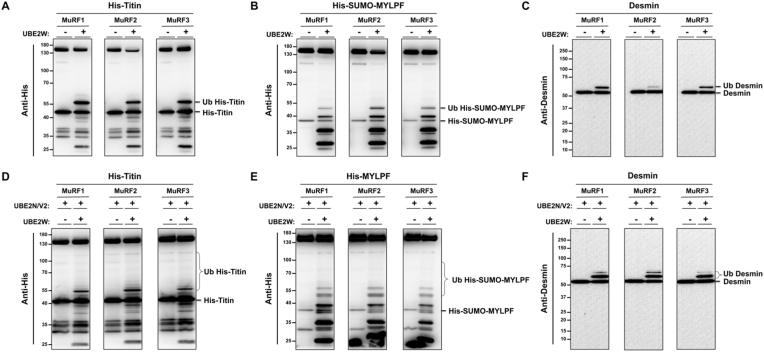
**MuRF1, MuRF2 and MuRF3 all target Titin, MYLPF and Desmin for ubiquitylation.** A & D) His-Titin (A168-A170), B & E) His-SUMO-MYLPF and C & F) Desmin were incubated with MBP-MuRF1, MBP-MuRF2 or MBP-MuRF3 with or without UBE2s (W and N/V2) for 1 h during an in vitro ubiquitylation assay. Samples were subject to SDS-PAGE gel electrophoresis before western blot imaging. Anti-His antibody was used to detect His-Titin and His-SUMO-MYLPF and Anti-Desmin antibody was used to detect Desmin.

To provide further evidence for MuRF overlap during ubiquitylation, we next explored whether MuRF1, 2 and 3 can ubiquitylate the same substrates in vitro. The first substrate tested was Titin (A168-170) which is well established as a site of MuRF1 translocation and interaction [21,[35], [36], [37]], and more recently a binding site for MuRF2 and MuRF3 [38]. Additionally, we included MYLPF and Desmin as in vitro substrates, previously identified as potential MuRF1 substrates [29,39,40]. The ubiquitylation assays revealed that MuRF1, MuRF2 and MuRF3 partner with UBE2W to ubiquitylate His-Titin (A168-A170) (Fig. 4A), His-SUMO-MYLPF (Fig. 4B) and Desmin (Fig. 4C), demonstrating them to be direct substrates of all MuRF E3 ligases. The two-step mechanism of UBE2W and UBE2N/V2 that had been identified during MuRF autoubiquitylation, is also seen with substrate ubiquitylation whereby UBE2N/V2 can only polyubiquitylated substrates once they have been monoubiquitylated by UBE2W (Fig. 4D, E and F). Notably, the polyubiquitylated substrates appeared more difficult to detect as each chain length gets diluted and subsequently becomes less sensitive to antibody detection. When blotting for His, we also detect His-tagged UBE1 and UBE2's, which also become ubiquitylated and appear near the predicted band for ubiquitylated His-Titin and His-SUMO-MYLPF (Fig. 4A, B, D and E). To confirm that the labelled bands in the His blots were in fact ubiquitylated Titin and MYLPF and not other His-tagged proteins present in the reaction, we also blotted for the ubiquitin and MYLPF respectively. These western blots showed the bands above His-Titin (A168-A170) and His-SUMO-MYLPF, confirming that these bands are the ubiquitylated substrates (Figs. S3 and S4). To ensure that our model of substrate ubiquitylation was valid, we wanted to confirm that substrate ubiquitylation did not occur just because of their close proximity to ubiquitin and ubiquitin-regulating enzymes. Using our model, we also tested Valosin-containing protein (VCP) - another potential MuRF1 substrate [29], and found that it is not directly ubiquitylated by MuRF1 (Fig. S5). Therefore, our model does not cause all proteins to be ubiquitylated and so we can take more confidence that Titin, MYLPF and Desmin are direct substrates of MuRF1, MuRF2 and MuRF3.

### 3.5 UBE2N, W, and V2 gene expression increases following denervation of mouse skeletal muscle

**Fig. 5:**
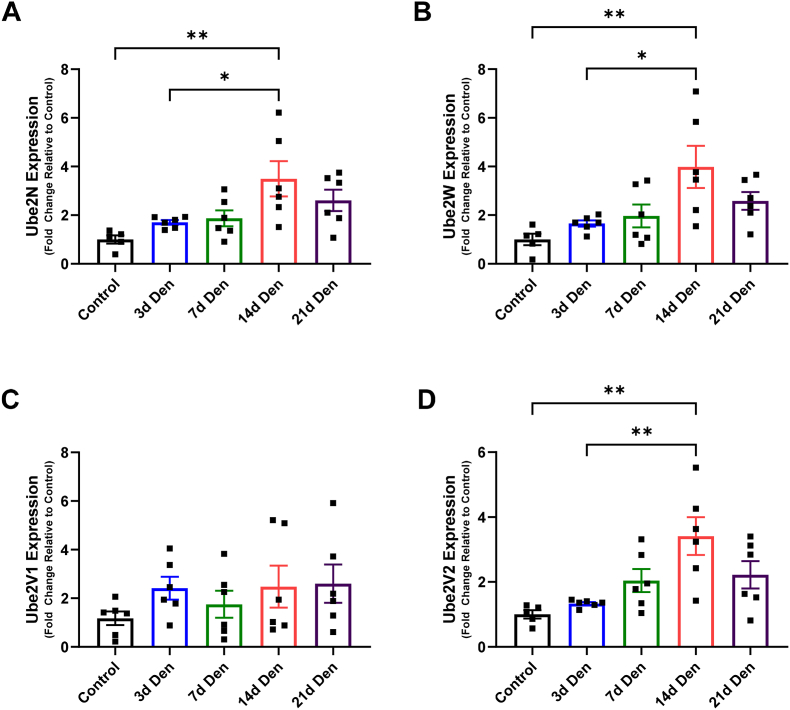
**UBE2N, UBE2W, UBE2V2, but not UBE2V1, mRNA expression increases following denervation of gastrocnemius complex muscle in mice.** C57BL/6 mice were grown for 3–4 months and then had their right leg denervated through surgical ablation of the sciatic nerve. Mice were sacrificed (at days 3, 7, 14, and 21) and their gastrocnemius UBE2 mRNA expression was measured by rt-qPCR. P-values were calculated using a one-way ANOVA with Tukey's post hoc test, *P < .05; **P < .01. Data presented as means ± SEM (n = 6).

Having identified MuRF E3 ligases cooperating with UBE2W and UBE2N/V during autoubiquitylation and substrate ubiquitylation, we wanted to investigate the importance of these UBE2s in skeletal muscle. MuRF1 is best known for its increased expression during different atrophic conditions, therefore we were interested to see if UBE2W and UBE2N/V followed a similar pattern. Using denervated mouse muscle that experienced muscle atrophy and increased MuRF1 mRNA expression [27], we measured the mRNA expression of UBE2W, UBE2N, UBE2V1 and UBE2V2. We confirmed the presence of all these UBE2's in muscle and found that UBE2W, UBE2N and UBE2V2 are upregulated following 14 days of denervation (Fig. 5A, B and D). Therefore, the two-step mechanism of UBE2W and UBE2N/V could play an important role in regulating the function of MuRF1 during denervation-induced muscle atrophy.

## 4 Discussion

In vitro ubiquitylation assays allow us to study direct reactions/interactions of ubiquitin-regulating enzymes. With this assay, we identified a list of UBE2s that can partner with MuRF1 during ubiquitylation. Further, we showed that MuRF1 partners with different UBE2s to form multiple chain types, including K48- and K63-linked polyubiquitin chains. We identified that MuRF1 can partner with UBE2W to monoubiquitylate itself (autoubiquitylation), which can then serve as an anchor for K63-linked polyubiquitylation. This two-step mechanism allows MuRF1 to directly ubiquitylate Titin (A168-A170), MYLPF and Desmin. Utilising this in vitro method, we also found that MuRF2 and MuRF3 share the same UBE2 partners as MuRF1 and can target the same substrates, providing a molecular explanation for their functional redundancy in vivo.

### 4.1 Sequential and overlapping mechanism in UBE2 interaction with MuRF1, MuRF2 and MuRF3

We identified that MuRF1 was able to partner with all the UBE2D and UBE2E members to form polyubiquitin chains. Previous research has already shown the capacity for MuRF1 to partner with UBE2D and E families to form polyubiquitin chains [16,19,20,41]. The mechanisms by which MuRF1 function with the UBE2D family are disputed, with yeast-two hybrid methods showing no physical interaction with UBE2D2 [42]. However, it is possible that UBE2 binding properties are altered when bound to ubiquitin, which is not included during these interaction-based methods. Therefore, it is important not to directly compare the findings of in vitro ubiquitylation studies to interaction-based studies. Our data highlights MuRF1 ubiquitylation activity through direct cooperation with the UBE2D and E family of enzymes and therefore aligns with the current literature. These two families of UBE2s make up a quarter of the ubiquitin-conjugating enzymes in humans, offering a substantial number of partners to facilitate MuRF1 activity.

Our data demonstrates a common familial function of MuRF1 with fellow TRIM E3s, TRIM21 and TRIM5α. Like these E3 Ligases, MuRF1 can form K63-linked polyubiquitin chains in a two-step fashion by partnering with UBE2N/V1, or V2 and UBE2W to form an anchor on the substrate [32,33]. It is crucial to understand the diversity of ubiquitin chain types generated by this mechanism, since chain types determine the fate of substrates. We were able to confirm that K48-linked polyubiquitin chains were not formed, however future work would benefit from screening all ubiquitin chain types. Having shown overlap of MuRF1-UBE2 partners with other TRIM E3 ligases, we hypothesised that this could occur with MuRF2 and MuRF3. Previous work using ELISA-based methods found MuRF1, MuRF2 and MuRF3 display autoubiquitylation activity with some individual UBE2s [16], here we demonstrated that MuRF1, MuRF2 and MuRF3 function with all the same UBE2 enzymes (UBE2Ds, Es, Ns/Vs and W) during in vitro ubiquitylation.

From a structural perspective, the reason for specific UBE2-MuRF interactions during ubiquitylation is likely due to the sequence of E3-binding surface present on UBE2 loop regions [12,13]. Sequence variations in these motifs therefore contribute to the specificity of E3 binding [43]. It is worth mentioning that the interaction strength between MuRF1 and UBE2's has been shown to increase in the presence of telethonin [15], so we cannot rule out the possibility that UBE2-MuRF1 binding would be altered in the presence of a substrate.

### 4.2 Overlap in MuRF1, MuRF2 and MuRF3 substrate ubiquitylation

Using our in vitro model, we demonstrated that Titin (A168-A170), MYLPF and Desmin are direct substrates of all MuRF E3 ligases. Recent work has alluded to MuRF1-dependent ubiquitylation of these substrates in cancer cachexia [44]. This demonstrates the importance of these substrates in MuRF1 ubiquitylation, however the importance of these substrates in MuRF2 and MuRF3 ubiquitylation is not known. Given that only MuRF1 plays a predominant role in targeting substrates for degradation during atrophy, it is possible that MuRF2 and MuRF3 contribute to the normal turnover of these cytoskeletal substrates. Studies have shown that MYLPF is degraded by MuRF1 after denervation-induced atrophy [40] and is reduced following 14 days of MuRF1 overexpression [29]. We found that MuRF1 directly ubiquitylates MYLPF, supporting the hypothesis that degradation of MuRF1 substrates is caused by direct ubiquitylation. By revealing that MuRF2 and MuRF3 can also ubiquitylate MYLPF, they may also contribute its degradation. Desmin has been previously shown to be degraded during fasting-induced atrophy in a TRIM32 dependent manner [45]. Their study showed that TRIM32 expression did not change by fasting-induced atrophy, whereas MuRF1 expression increased. Since revealing Desmin as a direct substrate of MuRF1, this raises the question whether both MuRF1 and TRIM32 are required to contribute to Desmin ubiquitylation, facilitating its degradation. MuRF2 may have a more protective role for Desmin ubiquitylation given its function for maintaining intermediate filament proteins [46]. In support, increased Desmin ubiquitylation was associated with increased protein abundance following ASB2β overexpression in which MuRF2 was also increased [47]. This study along with others have revealed 10 different ubiquitylation sites on Desmin [48]. Future work could investigate whether any of these sites are ubiquitylated by all MuRF E3 ligase which would imply some redundant role.

We showed that substrate ubiquitylation can occur by a two-step ubiquitylation with UBE2W and UBE2N/V enzymes. This offers a mechanistic link to previous work showing that MuRF E3 ligases regulate K63-linked ubiquitin chains on Titin to signal for the degradation via autophagy [38]. A recent study reported that MuRF1 ubiquitylation of Titin resulted in recruitment of NBR1 and P62, proteins that facilitate the autophagy of large protein cargo [49]. MuRF2 also interacts with NBR1/P62 on Titin [50]. These data build a causal chain of events that end with Titin autophagy by p62 and NBR1, mediated by K63-linked chains, instigated by the MuRF-UBE2 partners that we have elucidated.

### 4.3 Implications in muscle atrophy

Given the role of MuRF1 during muscle atrophy, we wanted to test whether the UBE2's they function with are also upregulated during muscle atrophy. In dexamethasone treated mice, UBE2E1 knockdown exacerbated muscle atrophy [51]. Therefore, UBE2E family may be important for protecting MuRF1 substrates from degradation. Furthermore, transcriptomics of mice treated with dexamethasone for 14 days have shown increased expression of UBE2D2, D3, N, V1, and V2 [52]. The UBE2D family are highly promiscuous, interacting with most E3's to form all ubiquitin-linkage types, as such the biological importance of increased UBE2D expression is difficult to address. Following denervation-induced atrophy in mice, we found that mRNA expression of UBE2N, W, and V2 transiently increased at 14 days post-denervation. However, the peak mRNA expression of these UBE2 enzymes occurs later than MuRF1 mRNA, which occurs 3–7 days post denervation [27]. The loss of muscle mass is significantly greater after 14 days denervation when compared to 3–7 days, which could suggest that these UBE2's only increase in response to more severe muscle wasting. The delayed transient expression of UBE2s shown in our work highlights the importance of screening multiple time points of atrophy when studying UBE2s. In the perspectives of drug discovery, disrupting MuRF1-E2 interactions at their peak expression could offer an efficient therapeutic approach to diminish MuRF1's effect on muscle atrophy.

## 5 Conclusion

**Fig. 6:**
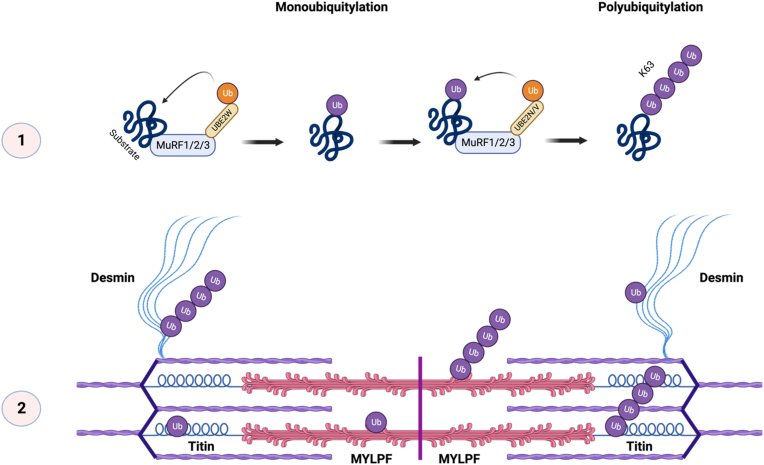
**Schematic of proposed explanation for MuRF E3 ligase functional redundancy.** 1) MuRF3 E3 ligases bind with UBE2W to transfer a single ubiquitin onto the substrate (monoubiquitylation). This ubiquitin modification acts as an anchor for K63-linked ubiquitin chain formation (polyubiquitylation) by UBE2N/V. 2) We propose that each MuRF E3 ligase can perform the above mechanism of ubiquitylation on Titin (A168-A170), MYLPF and Desmin. Created with BioRender.com.

The data presented here offers molecular insight into MuRF-induced ubiquitylation. We show that MuRF1 can interact with the UBE2D, E, N/V families and UBE2W during ubiquitylation. MuRF1 forms monoubiquitylation and creates K48- and K63-linked polyubiquitin chains in a UBE2 dependent manner. We provide evidence of direct substrates of MuRF1 ubiquitylation, namely Titin, MYLPF, and Desmin. Additionally, we show that these mechanisms are not specific to MuRF1, but also occur with MuRF2 and MuRF3. We propose that these findings are important for partial MuRF functional redundancy in vivo. Therefore, we have put forward a working hypothesis that under basal conditions MuRF1, MuRF2 and MuRF3 are co-ordinately involved in the ubiquitylation of certain substrates to ensure that individual loss of MuRF E3 ligase function does not impair the function of skeletal muscle (Fig. 6).

## Funding

S.L. was supported by the 10.13039/501100000268Biotechnology and Biological Sciences Research Council (BBSRC) and University of Birmingham-funded Midlands Integrative Biosciences Training Partnership (MIBTP) (BB/T00746X/1). P.D. was supported by MRC Versus Arthritis Centre for Musculoskeletal Aging Research (MR/P021220/1). J.C–O. was supported by 10.13039/501100016204Ministry of Higher Education, Science, Research and Innovation, Thailand and Department of Applied Thai Traditional Medicine, Faculty of Medicine, 10.13039/501100005790Thammasat University, Thailand. Y–C.L. was supported by MRC Versus Arthritis Centre for Musculoskeletal Aging Research (MR/P021220/1).

## CRediT authorship contribution statement

**Samuel O. Lord:** Writing – review & editing, Writing – original draft, Visualization, Methodology, Investigation, Conceptualization. **Peter W.J. Dawson:** Writing – original draft, Visualization, Methodology, Investigation, Conceptualization. **Jitpisute Chunthorng-Orn:** Writing – review & editing, Resources. **Jimi Ng:** Visualization, Writing – review & editing. **Leslie M. Baehr:** Visualization, Methodology, Investigation, Formal analysis. **David C. Hughes:** Writing – review & editing, Visualization, Methodology, Investigation, Formal analysis. **Pooja Sridhar:** Resources. **Timothy Knowles:** Supervision. **Sue C. Bodine:** Supervision. **Yu-Chiang Lai:** Writing – review & editing, Writing – original draft, Visualization, Supervision, Methodology, Investigation, Conceptualization.

## Declaration of competing interest

We declare no conflicts of interest, financial or otherwise.
