# Supplementary material for: Uncovering the mechanisms of MuRF1-induced ubiquitylation and revealing similarities with MuRF2 and MuRF3
Source: Biochem Biophys Rep. 2024 Jan 6;37:101636. doi: 10.1016/j.bbrep.2023.101636 (PMC10818185; doi:10.1016/j.bbrep.2023.101636)
Supplement: Multimedia component 1 [file mmc1.docx]

**Supplementary Material**

**
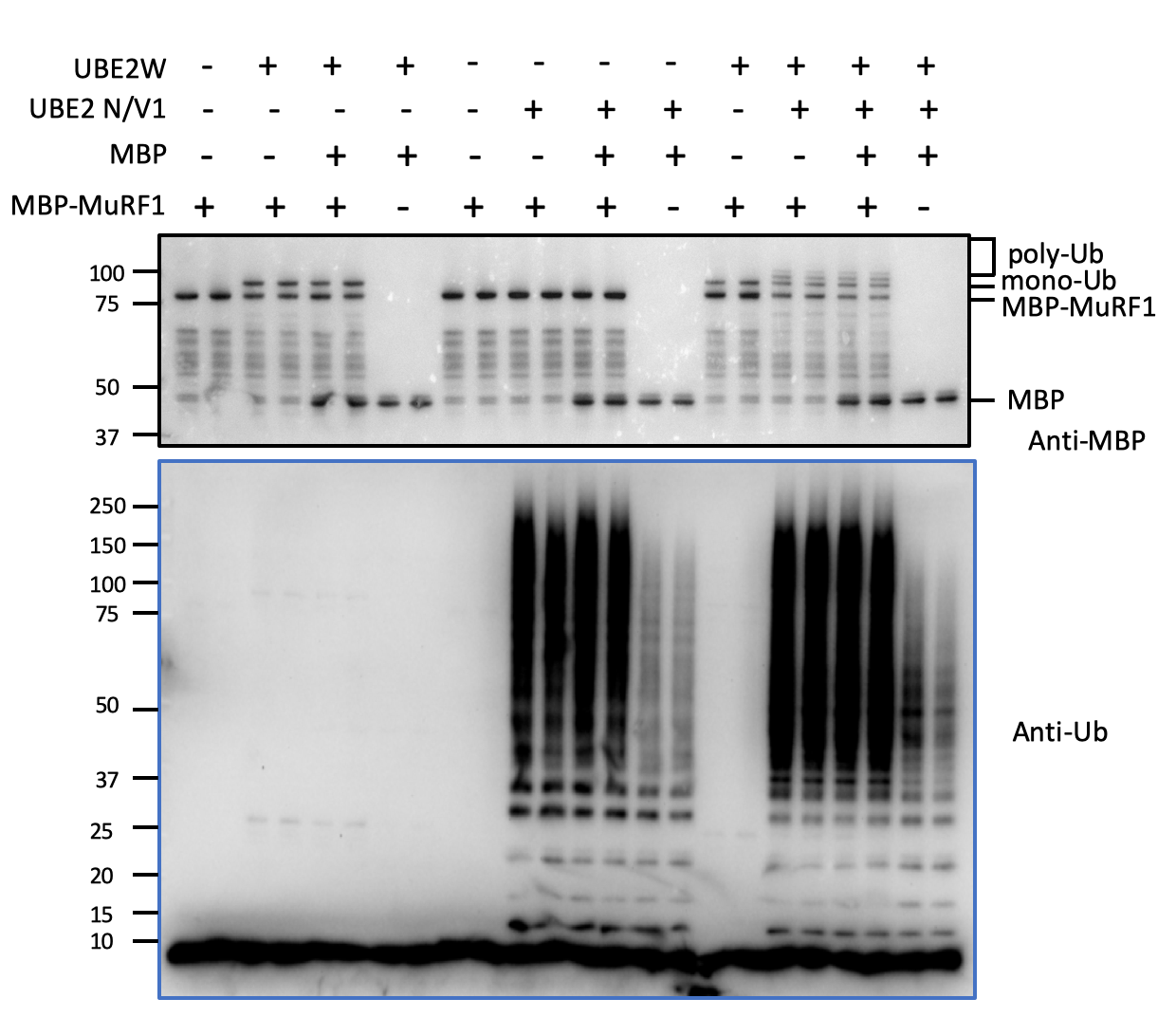
**

***Supplementary Figure 1.*** ***Ubiquitylation does not occur on MBP tag.*** *UBE2W, UBE2N/V1 or UBE2W + UBE2N/V1 were incubated with MBP-MuRF1 or MBP alone for 1 hour during an in vitro ubiquitylation assay. Each reaction was performed in duplicate and samples were subject to SDS-PAGE gel electrophoresis in before western blot imaging. Anti-MBP antibody was used to detect auto-ubiquitylation. Anti-ubiquitin antibody was used to detect ubiquitin chain formation.*


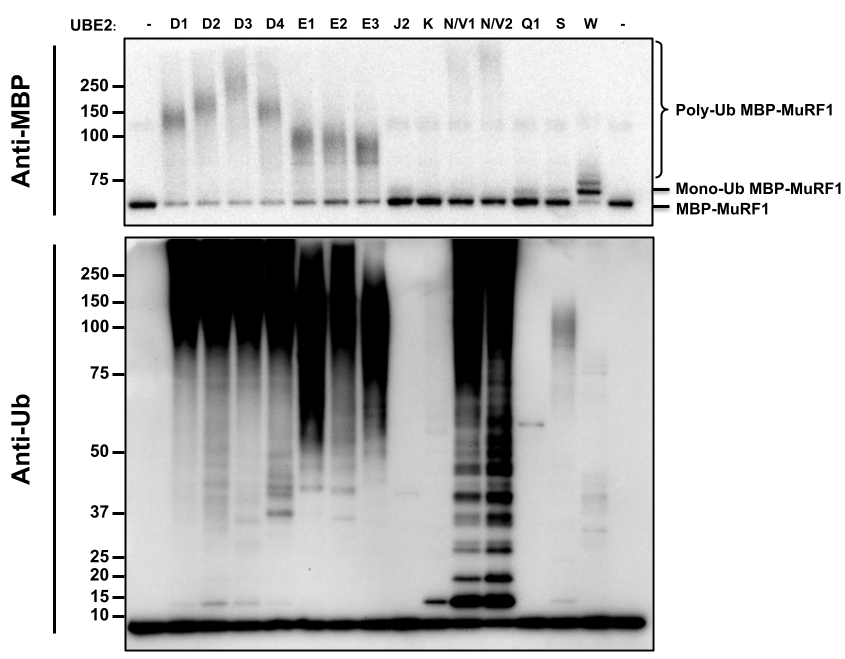


***Supplementary Figure 2.*** ***UBE2D, E and W family partner with MuRF1 to form autoubiquitylation, whereas UBE2N/V forms unanchored ubiquitin chains.*** *Ten selected UBE2s identified as MuRF1 partners (Fig 1) and four non-partners (UBE2J2, K, Q1, and S) as negative controls, were incubated with MBP-MuRF1 for 1 hour during an in vitro ubiquitylation assay. Samples were subject to SDS-PAGE gel electrophoresis before western blot imaging. Anti-MBP antibody was used to detect MBP-MuRF1 auto-ubiquitylation. Anti-ubiquitin antibody was used to detect ubiquitin chain formation.*

**
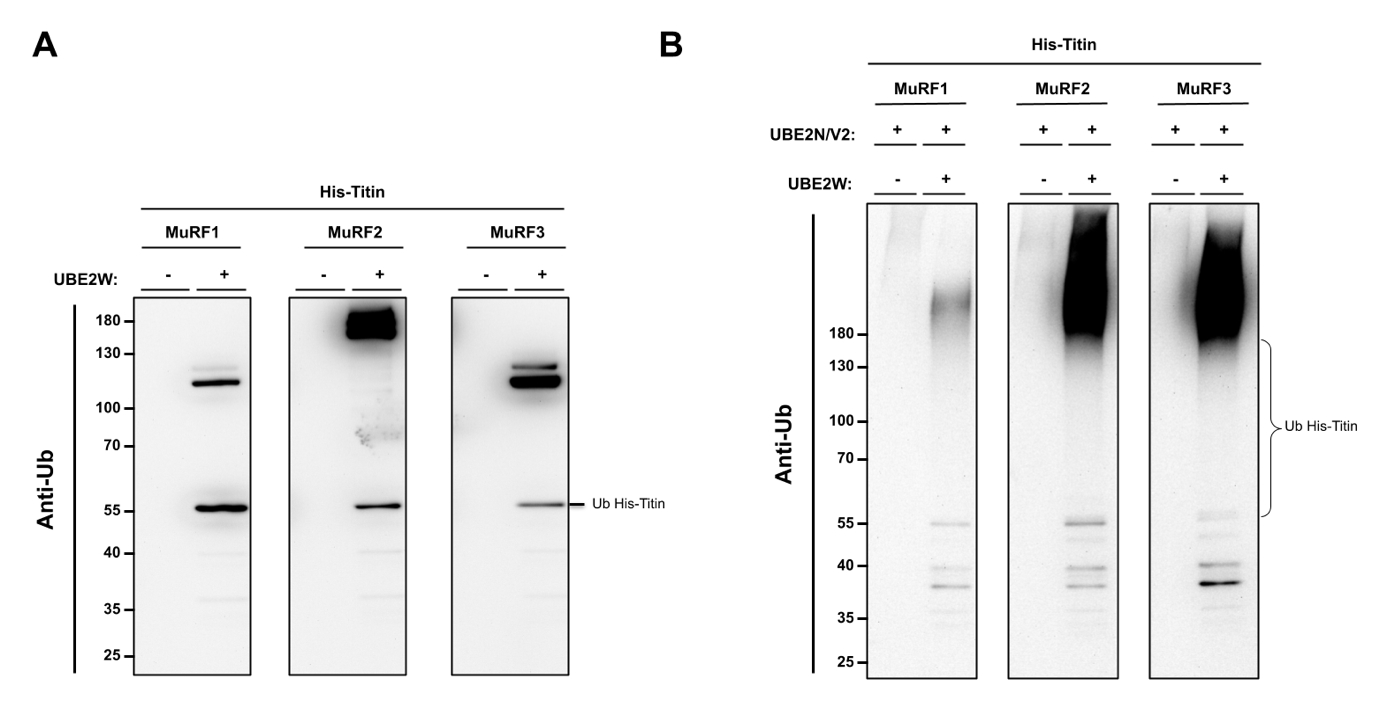
**

***Supplementary Figure 3: Anti-ubiquitin blot confirms Titin ubiquitylation by each MuRF E3 ligase.*** *His-Titin (A168-A170) was incubated with MBP-MuRF1, MBP-MuRF2 or MBP-MuRF3 with or without UBE2s (W or N/V2) for 1 hour during an in vitro ubiquitylation assay. Samples were subject to SDS-PAGE gel electrophoresis before western blot imaging. Anti-ubiquitylated proteins antibody was used to detect Titin monoubiquitylation (A) and Titin polyubiquitylation (B). The strong bands at the top of the membrane are autoubiquitylated MBP-tagged MuRF E3 ligases.*

***Supplementary Figure 4: Anti-MYLPF blot confirms MYLPF ubiquitylation by each MuRF E3 ligase.*** *His-SUMO-MYLPF was incubated with MBP-MuRF1, MBP-MuRF2 or MBP-MuRF3 with or without UBE2s (W or N/V2) for 1 hour during an in vitro ubiquitylation assay. Samples were subject to SDS-PAGE gel electrophoresis before western blot imaging. Anti-MYLPF antibody was used to detect MYLPF monoubiquitylation (A) and MYLPF polyubiquitylation (B).*

**
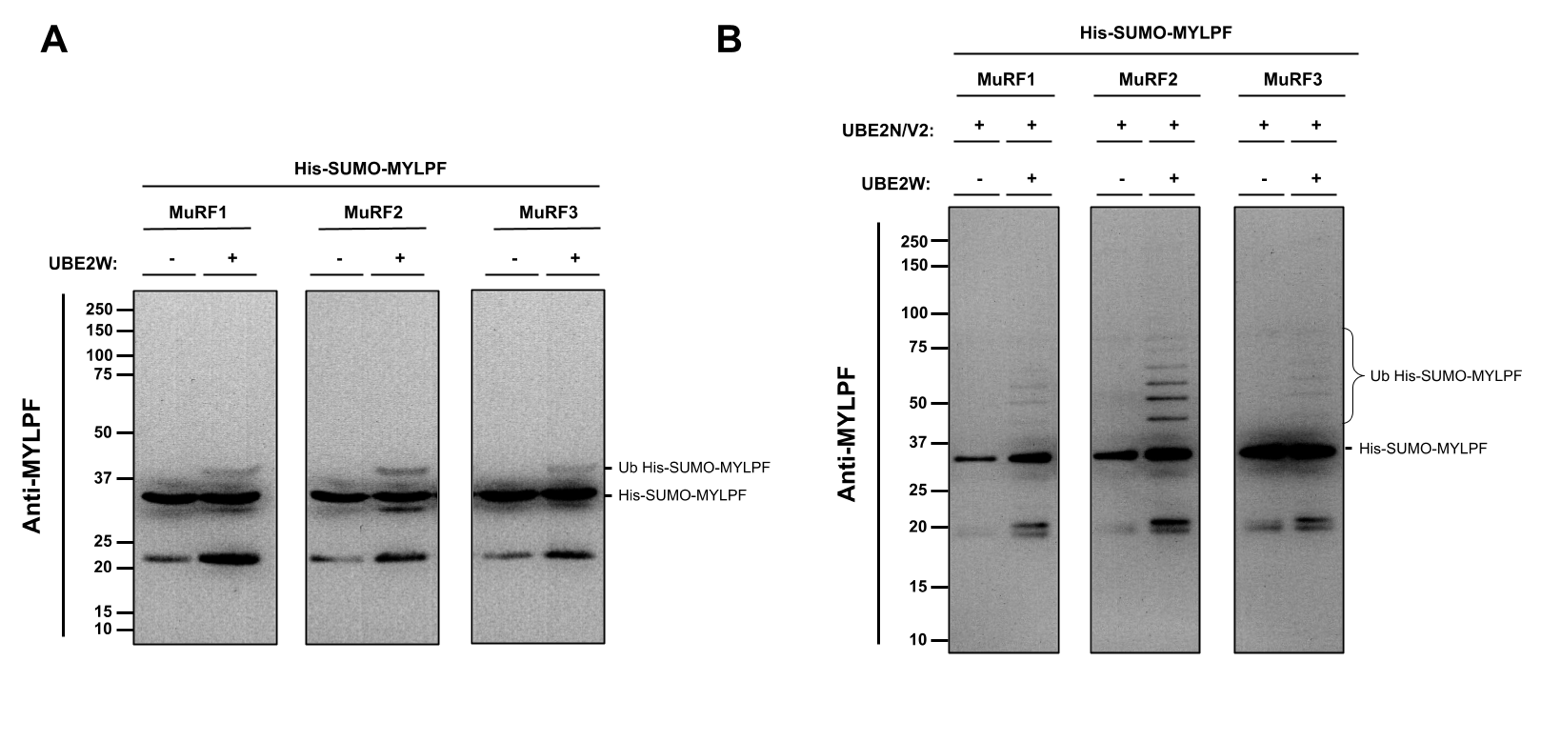
**


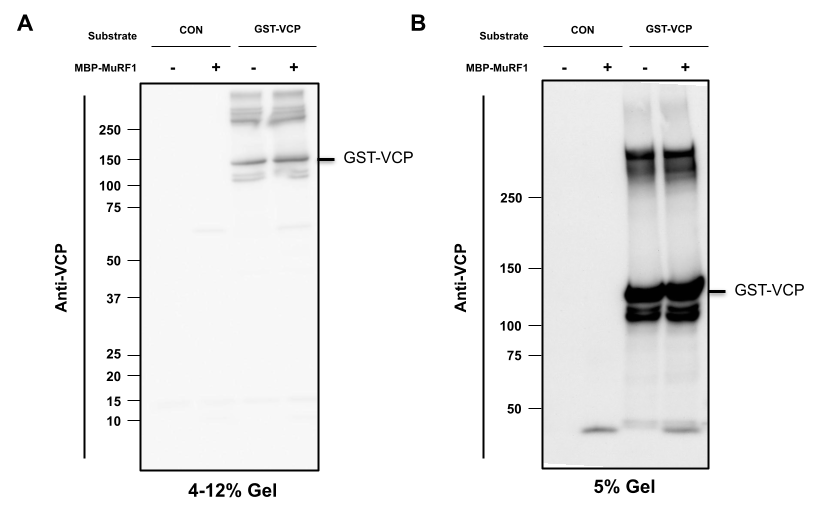


***Supplementary Figure 5: MuRF1 does not directly ubiquitylated VCP.*** *GST-VCP was incubated with or without MBP-MuRF1 for 1 hour during an in vitro ubiquitylation assay using UBE2W. Samples were subject to 4-12% acrylamide (A) or 5% acrylamide (B) SDS-PAGE gel electrophoresis before western blot imaging. Anti-VCP antibody was used to detect presence or absence of monoubiquitylated GST-VCP.*

**Supplementary Table 1 – UBE2s excluded from in vitro screening.**

| **Potential UBE2** | **Reason for exclusion** |
| --- | --- |
| UBE2F | NEDD8-conjugating E2 |
| UBE2I | SUMO-conjugating E2 |
| UBE2L6 | ISG15-conjugating E2 |
| UBE2M | NEDD8-conjugating E2 |
| UBE2QL | Only a probable E2 |
| BIRC6 | Chimeric E2/E3 |
| UBE2U | Only expressed in Testis |

**Supplementary Table 2 – Primers used for qPCR.**

| **Primers** | **Sequence (5’-3’)** |
| --- | --- |
| Trim63 (MuRF1)-F | GCTGGTGGAAAACATCATTGACAT |
| Trim63 (MuRF1)-R | CATCGGGTGGCTGCCTTT |
| UBE2N-F | CCAATGGCAGCACCTAAAGTACG |
| UBE2N-R | GGATTGATAGCAGAACTGTGCGG |
| UBE2W-F | CGATACCCTTTTGACTCTCCTCA |
| UBE2W-R | TGCTGAGACAGACTGACTGCAC |
| UBE2V1-F | GATAGAGTGTGGGCCTAAGTACC |
| UBE2V1-R | GAGTTCTGCCACTTTGCCAGCA |
| UBE2V2-F | TACCCAGAAGCTCCTCCATCAG |
| UBE2V2-R | GCTAATACTGGTATGCTCCGTGC |

**Supplementary Table 3. List of plasmids used for generating recombinant proteins.** Plasmids obtained from Medical Research Council – Protein Phosphorylation and Ubiquitylation Unit Reagents and Services (<https://mrcppureagents.dundee.ac.uk>) are listed, while others are detailed in the Methods.

| **Plasmid** | **Protein name** | **Identity (DU number)** |
| --- | --- | --- |
| pFastBac HTb 6His TEV UBE1 | UBE1 | DU32888 |
| pGEX6P-3-GST-UBE2A | UBE2A | DU4203 |
| pET156P-1 6His UBE2B | UBE2B | DU32555 |
| pET156P 6His UBE2C | UBE2C | DU32146 |
| pET28a(+) 6His-UBE2D1 (UBCH5a) | UBE2D1 | DU4315 |
| pET28- 6His-UBE2D2 | UBE2D2 | DU20184 |
| pET156P 6His 3C UBCH5c | UBE2D3 | DU15703 |
| pET28a 6His-UBE2D4 | UBE2D4 | DU8232 |
| pET156P 6His-UBCH6 | UBE2E1 | DU12803 |
| pET28a 6His-UBE2E2 | UBE2E2 | DU12394 |
| pET28a(+)6His-UBE2E3 | UBE2E3 | DU14049 |
| pET28a(+)6His-UBE2G1 | UBE2G1 | DU14055 |
| pET28a 6His-UBE2G2 | UBE2G2 | DU20174 |
| pET156P 6His-UBE2H | UBE2H | DU32149 |
| pET28a 6His-UBE2J1 1-282 | UBE2J1 | DU20686 |
| pET28a 6His-UBE2J2 1-226 | UBE2J2 | DU20695 |
| pET156P 6His-UBC1 | UBE2K | DU20018 |
| pET156P 6His-3C-UBCH7 | UBE2L3 | DU12798 |
| pET15b 6His-3C-UBC13 | UBE2N | DU15705 |
| pET28a 6His-UBE2V1 | UBE2V1 | DU20179 |
| pET15b 6His-C3-UBE2V2 | UBE2V2 | DU12415 |
| pET156P 6His-UBE20 | UBE2O | DU32152 |
| pET28 6His-UBE2Q1 | UBE2Q1 | DU20176 |
| pET15b 6His-3C-UBE2Q2 | UBE2Q2 | DU12801 |
| pET28a(+) 6His-CDC34 | UBE2R1 | DU4317 |
| pGEX6P-1-GST-UBE2R2 | UBE2R2 | DU4616 |
| pET28-6His-UBE2S | UBE2S | DU20175 |
| pET15b6P 6His-UBE2T | UBE2T | DU12416 |
| pET28a-6His-UBE2W isoform 1 | UBE2W | DU20190 |
| pET15b 6HIS C3 6His-UBE2Z | UBE2Z | DU20121 |
| pMEX3Cb MBP 3C-TEV-TRIM63 | MBP-MuRF1 | DU58528 |
| pMEX3Cb MBP-TRIM54 | MBP-MuRF3 | DU49175 |
| pMEX3Cb MBP-TRIM55 | MBP-MuRF2* | uncatalogued |
| pET24 Ubiquitin | Ubiquitin | uncatalogued |

*TRIM55 gene (pcDNA3.1) was purchased from GenScript (Clone ID OHu29713) and then shuttled into pMEX3Cb MBP-tag vector
