# Supplementary material for: Uncovering the mechanisms of MuRF1-induced ubiquitylation and revealing similarities with MuRF2 and MuRF3
Source: Biochem Biophys Rep. 2024 Jan 6;37:101636. doi: 10.1016/j.bbrep.2023.101636 (PMC10818185; doi:10.1016/j.bbrep.2023.101636)

**Raw uncropped WBs**

**“Ubiquitin for Fig 1”**

**
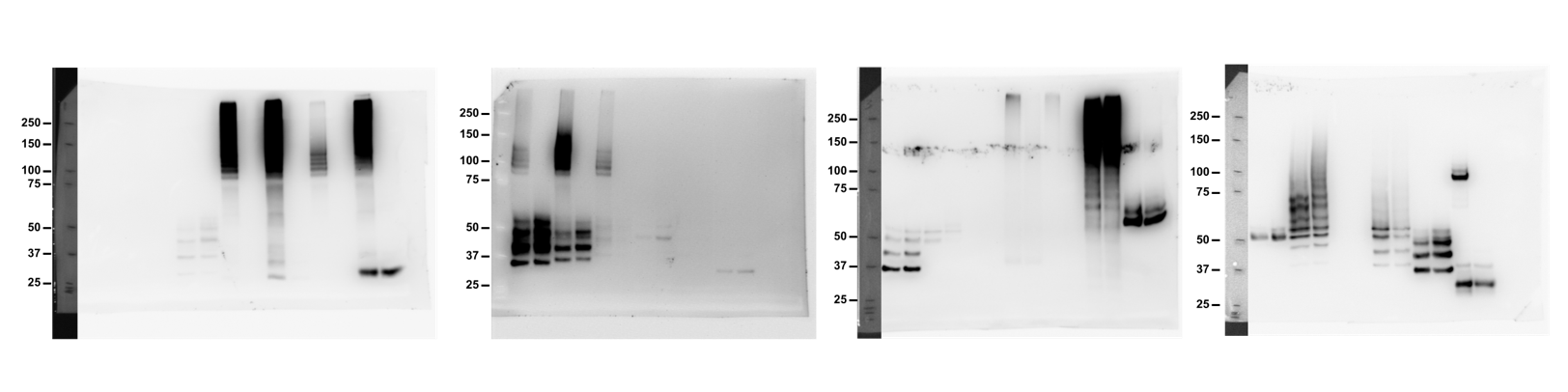
**

**“MBP for Fig 2A”**

**
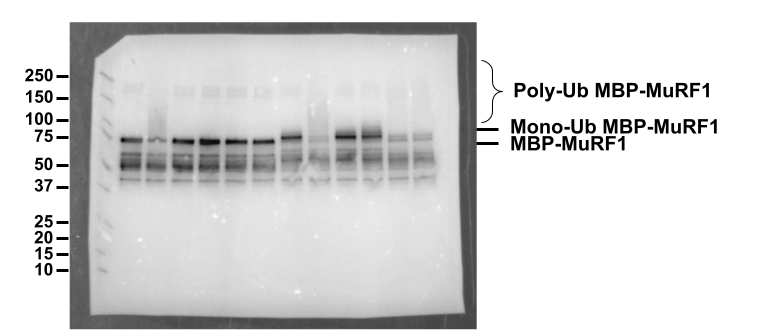
**

**“Ubiquitin for Fig 2B”**

**
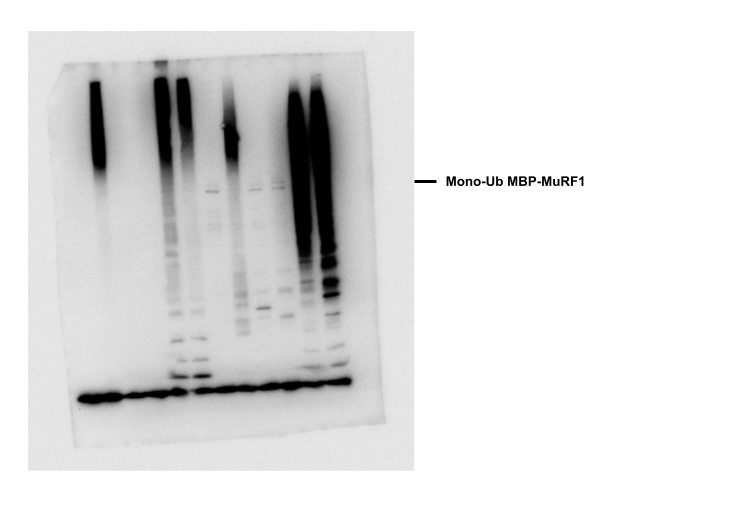
**

**
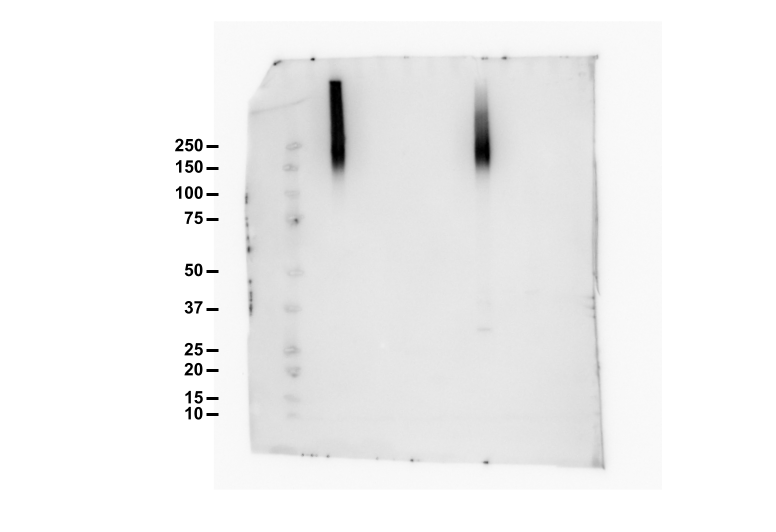
“K48 for Fig 2C”**

**“K63 for Fig 2C”**

**
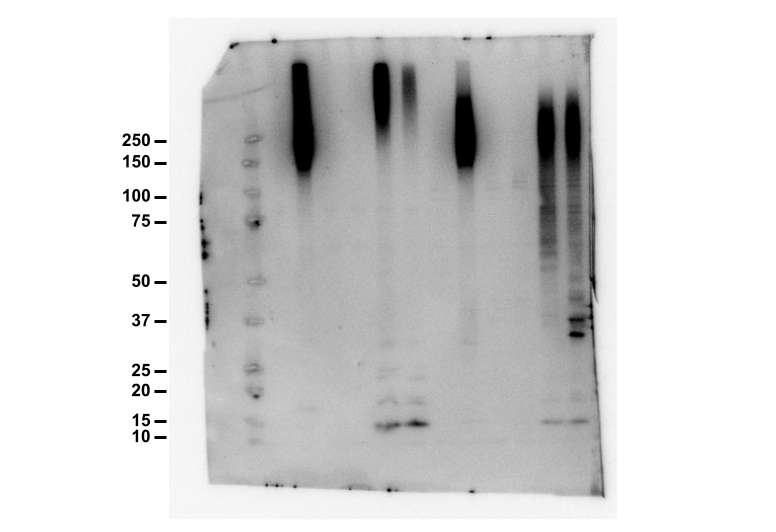
**

**“Ubiquitin for Fig 3A”**

**
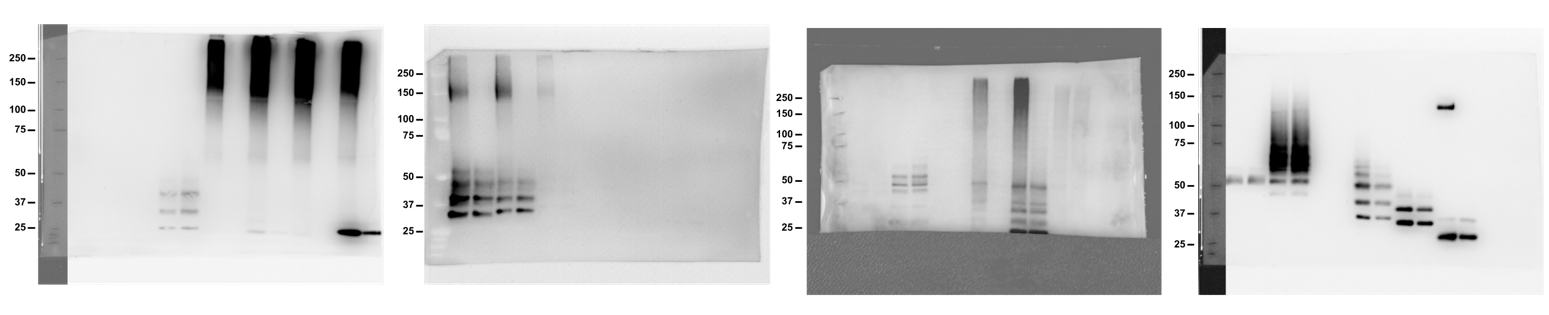
**

**“Ubiquitin for Fig 3B”**

**
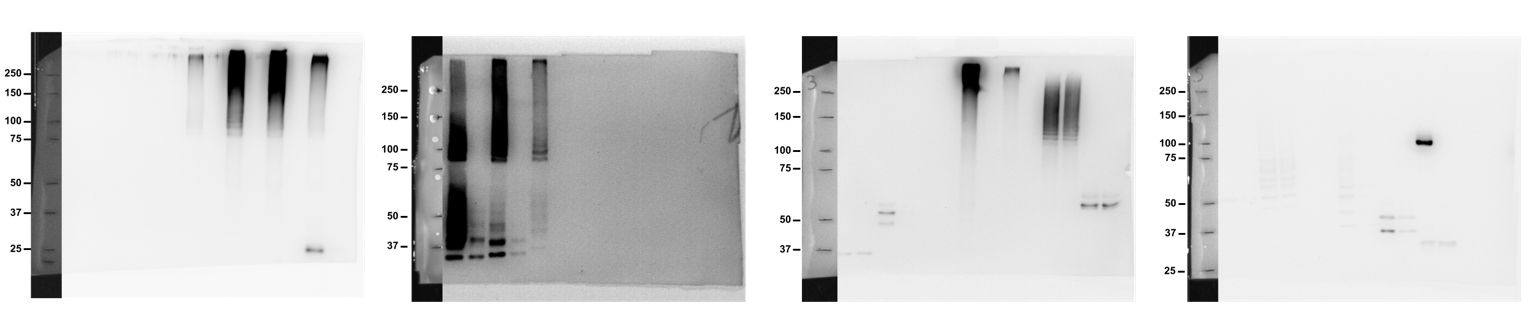
**

**“His for Fig 4A”**

Note: Fig 4A splits the image for each MuRF protein

**
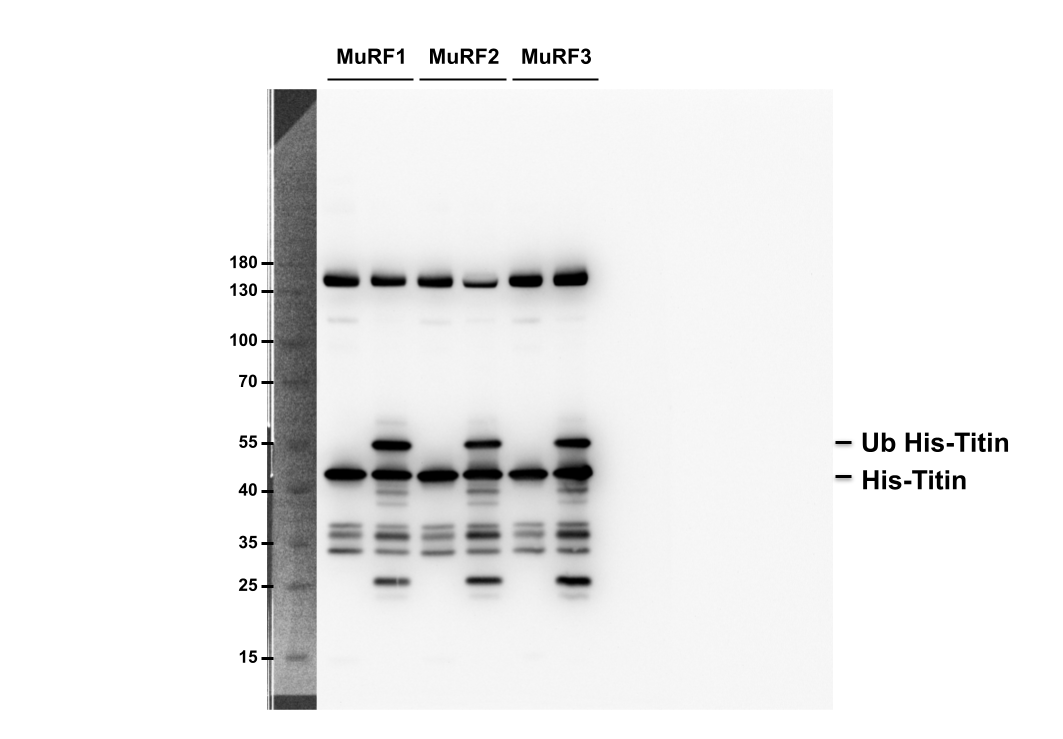
**

**“His for Fig 4B”**

Note: Fig 4B splits the image for each MuRF protein


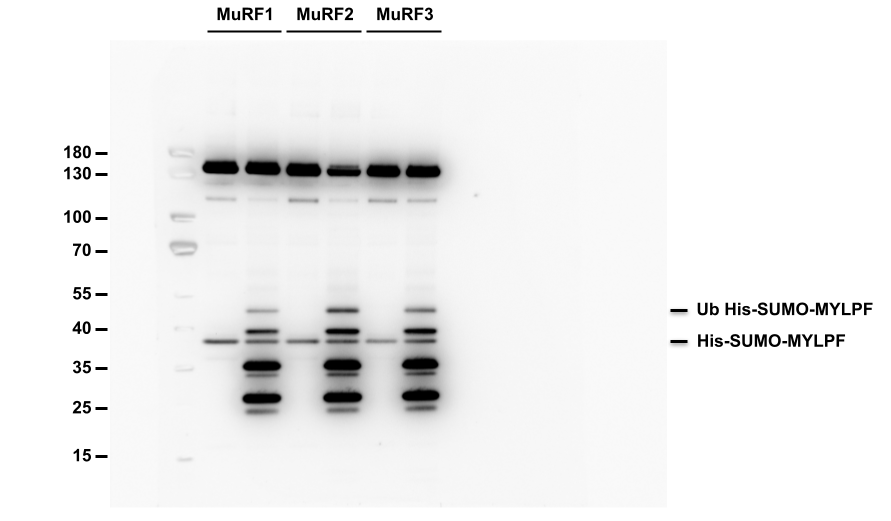


**“Desmin for Fig 4C and Fig 4F”**

Note: Fig 4C and Fig 4F splits the image for each MuRF protein

Also only Lanes “-“ and “W” are used for Fig 4C and only Lanes “ N/V2” and “W+N/V2” used for Fig 4F. Lane “D2” not used in figures


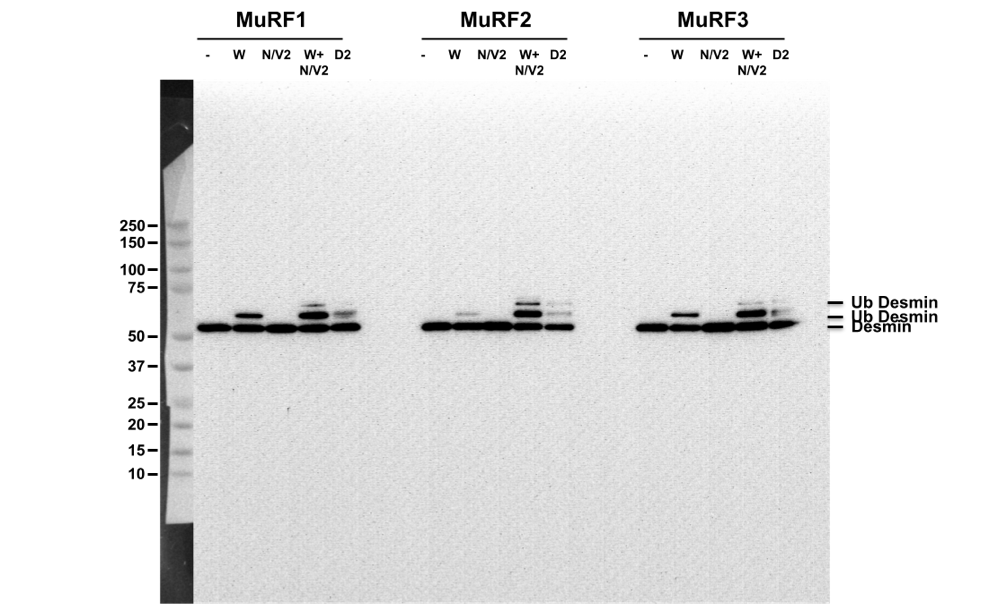


**“His for Fig 4D”**

Note: Fig 4B splits the image for each MuRF protein


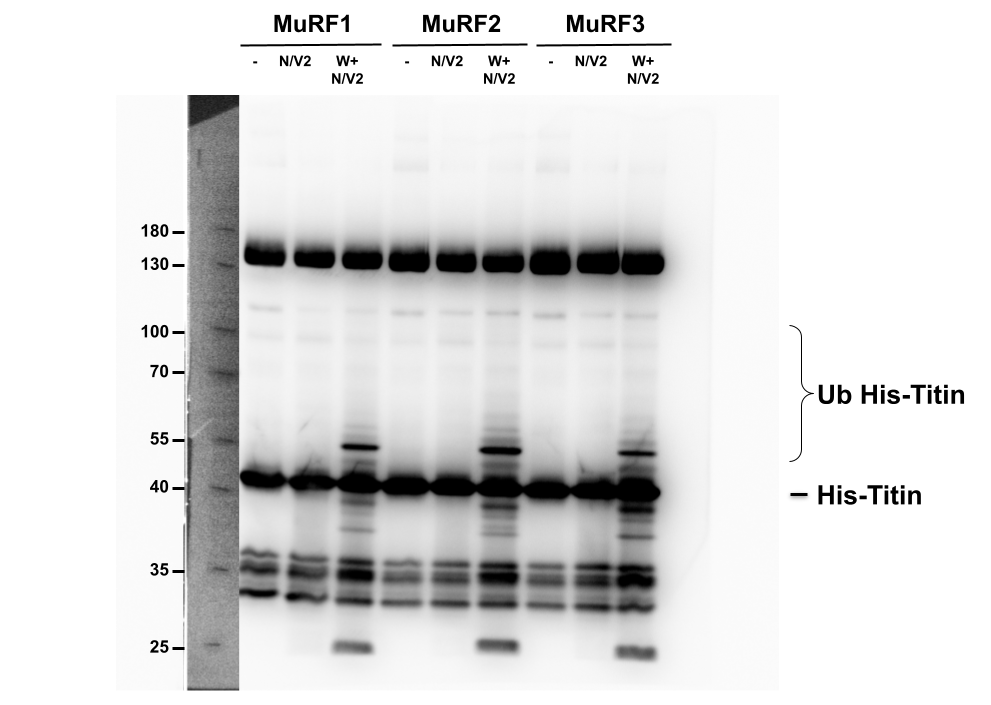
Also, Lanes “-” not used in figures

**“His for Fig 4E”**

Note: Lanes “-” not used in figures


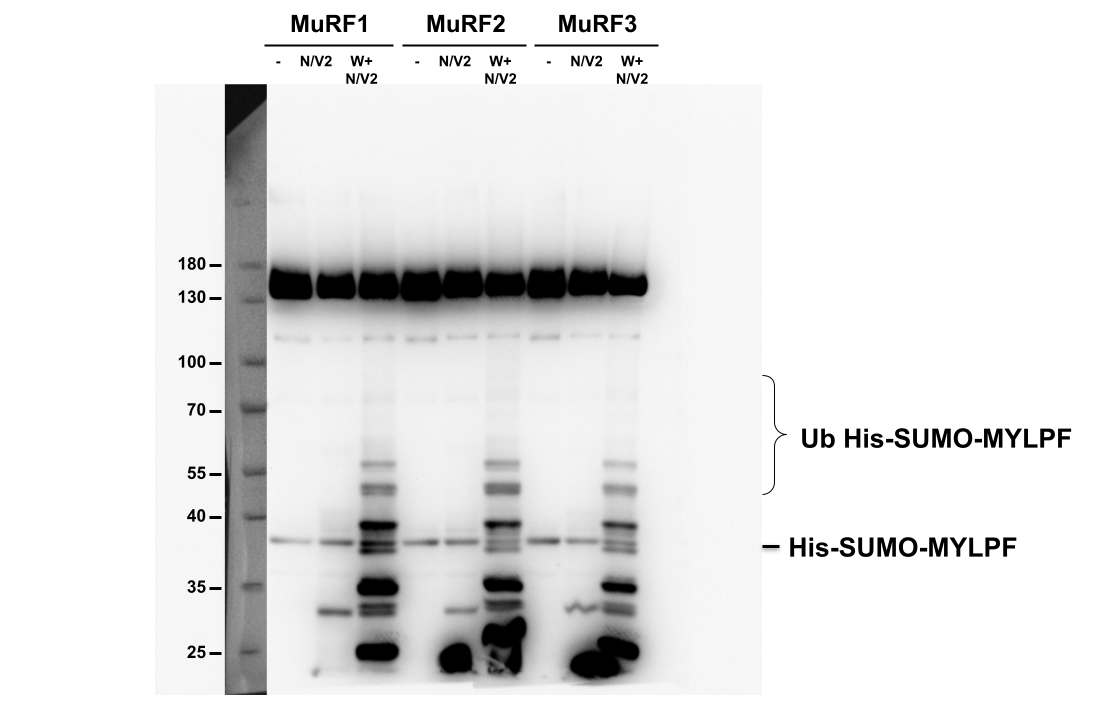


**“MBP for Fig S1”**
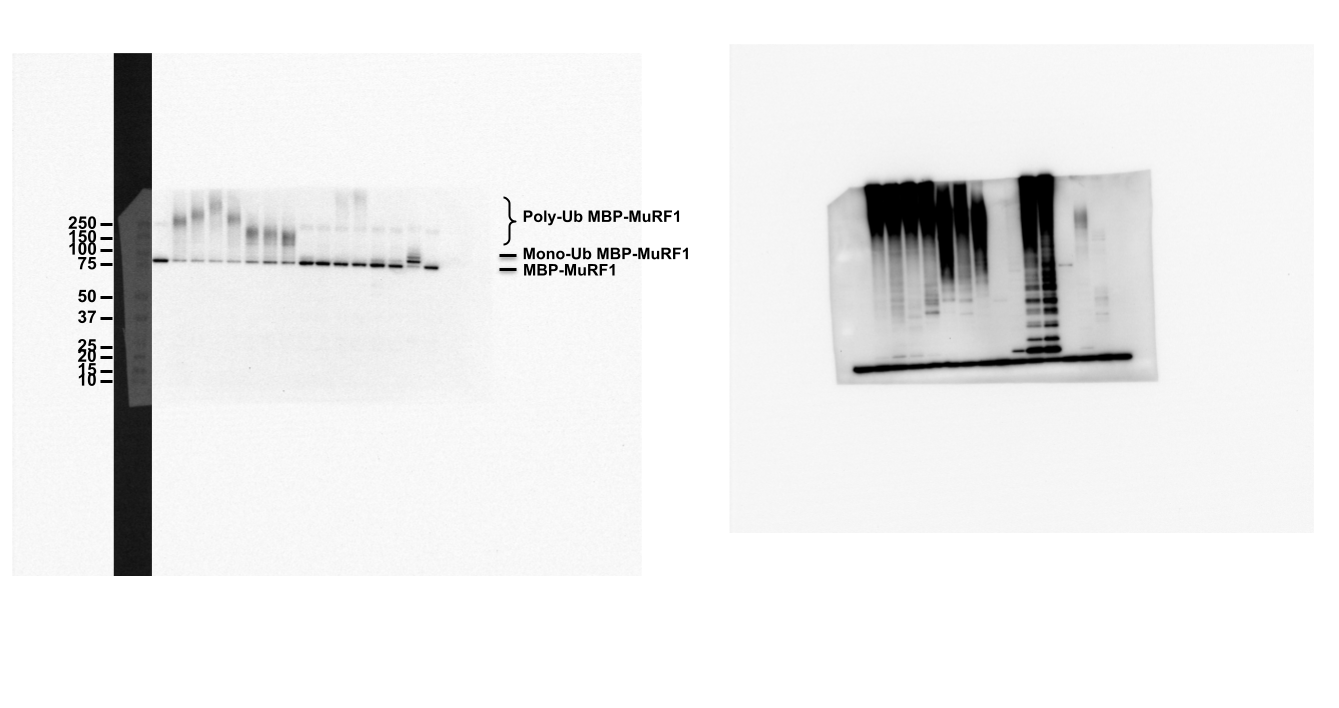
 **“Ubiquitin for Fig S1”**

**“Ubiquitin for Fig S2A”**

**
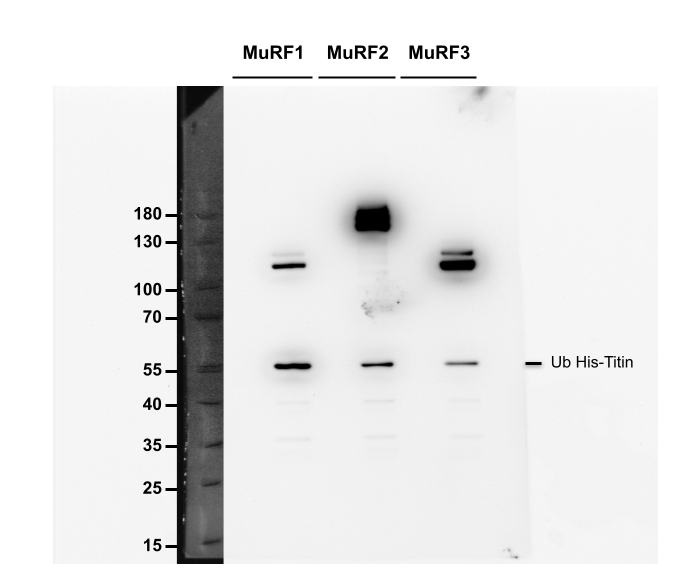
**Note: Fig S2 splits the image for each MuRF protein

**“Ubiquitin for Fig S2B”**

Note: Fig S2B splits the image for each MuRF protein

Also, Lanes “-” not used in figures


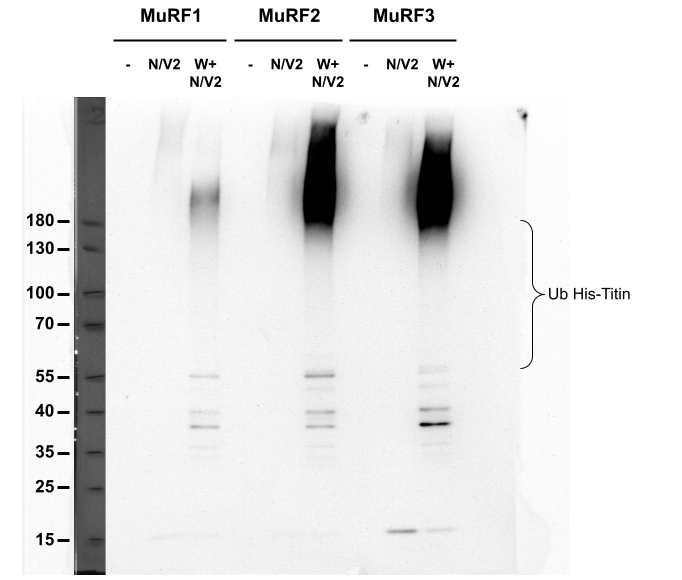


**“MYLPF for Fig S3A”**

Note: Fig S3A splits the image for each MuRF protein


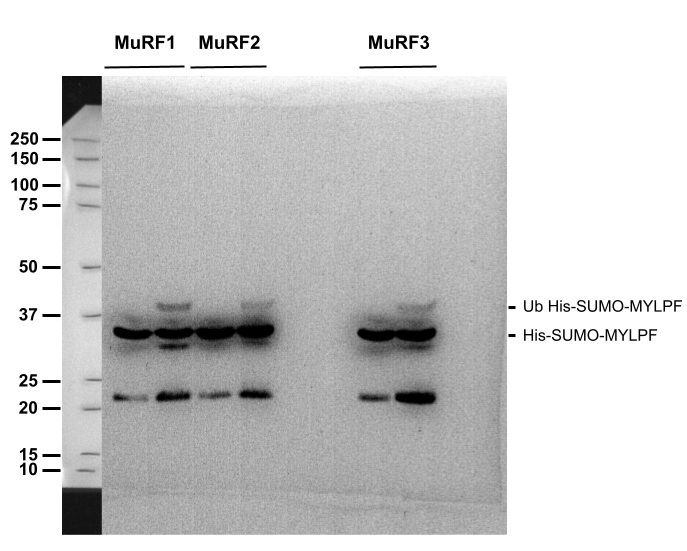


**“Ubiquitin for Fig S3B”**

Note: Fig S3B splits the image for each MuRF protein

Also, Lanes “-” not used in figures


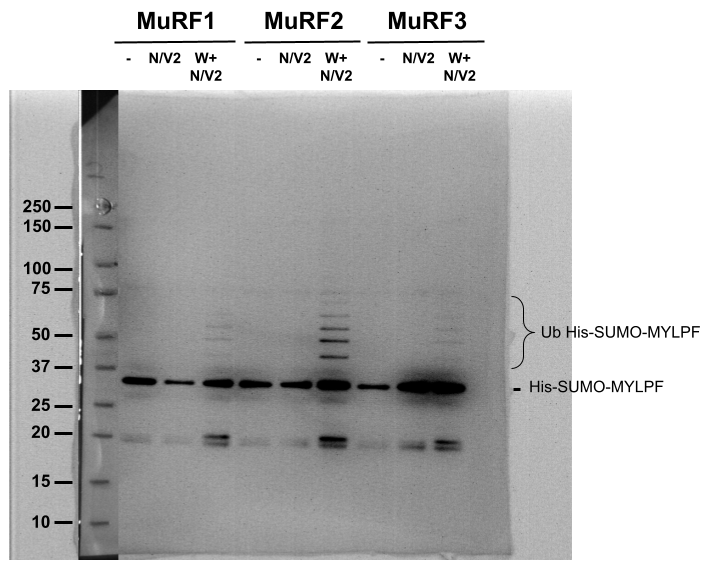


**“VCP for Fig S4A”**

**
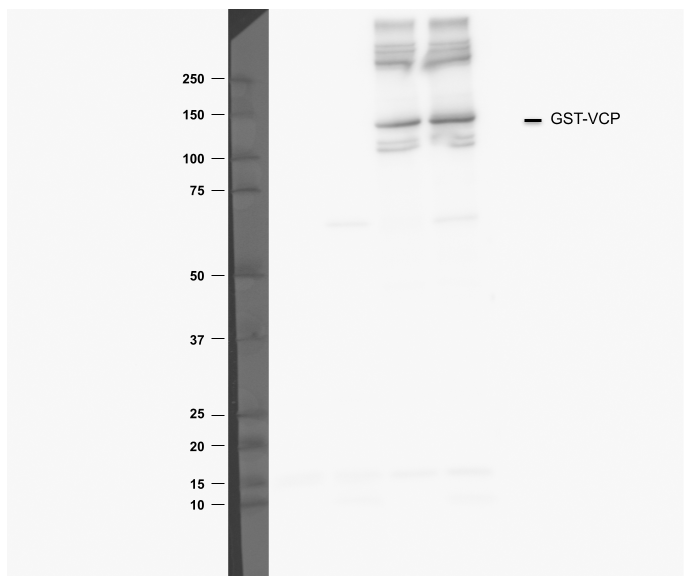
**

**“VCP for Fig S4B”**


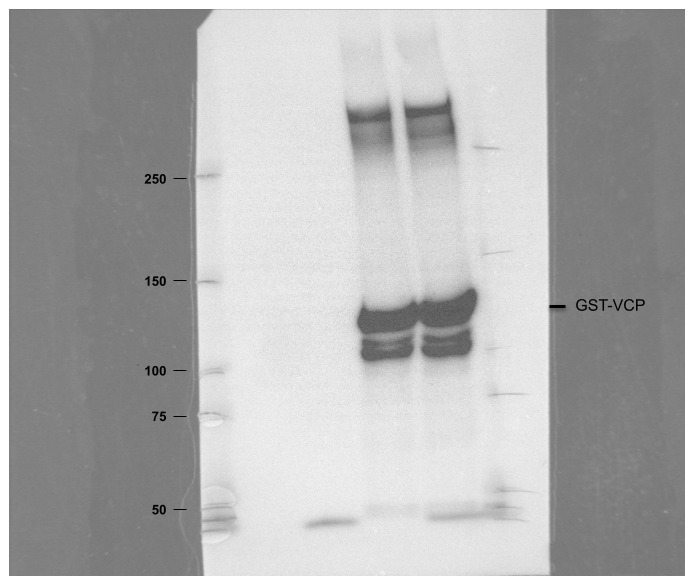

Supplement: Multimedia component 2 [file mmc2.docx]
